# Nonlinear Models of Thermo-Viscoelastic Materials

**DOI:** 10.3390/ma14247617

**Published:** 2021-12-10

**Authors:** Claudio Giorgi, Angelo Morro

**Affiliations:** 1 DICATAM, Università di Brescia, 25133 Brescia, Italy; claudio.giorgi@unibs.it; 2 DIBRIS, Università di Genova, 16145 Genova, Italy

**Keywords:** viscoelastic materials, viscoplastic materials, materials of stress-rate type, large-strain rate-dependent theories, thermodynamics, 74D10, 74C20, 74F05, 76A05, 76A10, 74A15, 80A17

## Abstract

The paper develops a general scheme for viscoelastic materials, where the constitutive properties are described by means of measures of strain, stress, heat flux, and their time derivatives. The constitutive functions are required to be consistent with the second law of thermodynamics. Indeed, a new view is associated with the second law: the non-negative expression of the entropy production is set equal to a further constitutive function. The introduction of the entropy production as a constitutive function allows for a much wider range of models. Within this range, a scheme to obtain nonlinear models of thermo-viscoelastic materials subject to large deformations is established. Notably, the Kelvin–Voigt, Maxwell, Burgers, and Oldroyd-B viscoelastic models, along with the Maxwell–Cattaneo heat conduction, are obtained as special cases. The scheme allows also for modelling the visco-plastic materials, such as the Prandtl–Reuss work-hardening function and the Bingham–Norton fluid.

## 1. Introduction

Viscoelasticity, especially the model of linear viscoelastic solids, traces back to Boltzmann [1], who, in essence, considered an elastic material with memory. The model elaborated by Boltzmann was based on the following assumptions.

At any point of the body, the stress at any time *t* depends upon the strain at all preceding times. If the strain up to time *t* is in the same direction, then the effect is to reduce the corresponding stress. The influence of a previous strain on the stress depends on the time elapsed since that strain occurred and is weaker for strains that occurred long ago. In addition, a superposition of the influence of previous strains holds, which means that the stress–strain relation is linear. Consequently, in the linear (infinitesimal) approximation, the Cauchy stress T is given by the infinitesimal strain ε in the form

$$T\left(t\right)=K_{0}\varepsilon \left(t\right)+\int_{0}^{\infty }K\left(s\right)\varepsilon \left(t-s\right)ds,$$

where $K_{0}$ and K(s) take values in the space of fourth-order tensors for any s≥0 while $T,K_{0},K$, and ε are considered at fixed points of the body. The function K on [0,∞) is called the Boltzmann function. To adhere to the standard notation, let G on [0,∞) be defined by

$$G\left(s\right)=G_{0}+\int_{0}^{s}K\left(\xi \right)d\xi ,\;G_{0}=K_{0},\;G_{\infty }=\lim_{s\to \infty }G\left(s\right);$$

G is called the relaxation function, $G_{0}$ is the instantaneous elastic modulus, and $G_{\infty }$ is the equilibrium elastic modulus. We can then write [2,3]

(1) $$T\left(t\right)=G_{0}\;\varepsilon \left(t\right)+\int_{0}^{\infty }G^{\prime }\left(s\right)\varepsilon \left(t-s\right)ds,$$

It is assumed that, for solids, both $G_{0}$ and $G_{\infty }$ and their difference $G_{0}-G_{\infty }$ are positive definite [2,4].

Rather than by memory functionals, viscoelastic properties can be modeled by rate (differential) equations [5,6]. For definiteness, let

$$G\left(s\right)=G_{0}\exp\left(-s/\tau \right).$$

Hence time differentiation—denoted by a superposed dot—of (1) yields

$$\begin{array}{cc} \dot{T} & =G_{0}\dot{\varepsilon }+\frac{1}{\tau }G_{0}\int_{0}^{\infty }\exp\left(-\frac{s}{\tau }\right)\frac{d\varepsilon (t-s)}{ds}ds\\ & =G_{0}\dot{\varepsilon }-\frac{1}{\tau }G_{0}\varepsilon +\frac{1}{\tau^{2}}G_{0}\int_{0}^{\infty }\exp\left(-\frac{s}{\tau }\right)\varepsilon \left(t-s\right)ds, \end{array}$$

whence we have the Maxwell model

(2) $$\dot{T}+\frac{1}{\tau }T=G_{0}\dot{\varepsilon }.$$

The purpose of this paper is to establish thermodynamically consistent models of viscoelastic materials characterized by rate (or differential) equations for stress and strain. The approach is quite general in that the constitutive equations are nonlinear and three-dimensional and involve finite deformations. Furthermore, the thermodynamic restrictions are determined so that coupling effects are allowed between stress rate, strain rate, and entropy production; formally, this is realized through a novel representation formula.

The memory effects of viscoelasticity are modeled jointly with thermal properties. In this respect, we observe that dissipative properties are ascribed to the materials models; there are approaches or models where dissipative effects are associated with external damping or boundary conditions [7].

Our approach is inherently thermodynamic. We derive constitutive vector- and tensor-valued rate equations consistent with thermodynamics in that they obey the second law (or Clausius–Duhem) inequality. This is the way we distinguish physically admissible models from purely mathematical ones, as is standard in rational thermodynamics. The pertinent variables of the constitutive equations comprise stress, strain, heat flux, and their time derivative.

It is a key point of our approach that the entropy production enters as a non-negative constitutive function. This in turn improves the generality of the materials models (see, for instance, [8]). Depending on the type of model, we proceed in the referential (Lagrangian) description or in the spatial (Eulerian) description. By arguing in the referential configuration, we obtain objective descriptions in the corresponding Eulerian description [9]. The main advantage of the present approach is the possibility of establishing nonlinear, thermodynamically consistent, objective rate-type relations describing memory and dissipative effects.

It is worth mentioning References [10,11], where viscoelastic models are developed in the relativistic Landau–Lifshitz frame on the basis of Onsager’s linear non-equilibrium thermodynamics. The models are based on the energy momentum tensor; the particle-number current; and where appropriate, the rheology equations. The constitutive equations are required to be consistent with the maximum property of the entropy functional in the process of linear regression. The maximum property of the entropy seems to be the analog of our requirement of non-negative entropy production. Instead, our scheme involves properties and processes such as nonlinearities and hysteretic effects [8,12,13].

## 2. Balance Laws and Entropy Inequality

We consider a body occupying the time-dependent region $\Omega \subset E^{3}$. The motion is described by means of the function χ(X,t), providing the position vector x∈Ω in terms of the position X, in a reference configuration R, and the time *t*, so that Ω=χ(R,t). The symbols $\nabla ,\nabla {\;}_{R}\;$ denote the gradient operator with respect to x∈Ω,X∈R. The function χ is assumed to be differentiable; hence, we can define the deformation gradient as $F=\nabla {\;}_{R}\;\chi$ or, in suffix notation, $F_{iK}=\partial_{X_{K}}\chi_{i}$. The invertibility of X↦x=χ(X,t) is guaranteed by letting J:=detF>0. Let v(x,t) be the velocity field, on $\Omega \times R^{+}$. A superposed dot denotes time differentiation following the motion of the body, and hence, for any function f(x,t), we have $\dot{f}=\partial_{t}f+v\cdot \nabla f$. We denote by L the velocity gradient, $L_{ij}=\partial_{x_{j}}v_{i}$ and recall that

$$\dot{F}=LF.$$

Moreover D denotes the stretching tensor, D=SymL, and W the spin tensor W=SkewL. In terms of F, the (right) Cauchy–Green tensor C and the Green–Saint Venant deformation tensor E are defined by

$$C:=F^{T}F,\;E:=\frac{1}{2}\left(C-1\right).$$

Let ε be the internal energy density (per unit mass), T be the symmetric Cauchy stress, q be the heat flux vector, ρ be the mass density, *r* be the (external) heat supply, and b be the mechanical body force per unit mass. The balance equations for mass, linear momentum, and energy are taken in the form

(3) $$\dot{\rho }+\rho \nabla \cdot v=0,\;\rho \dot{v}=\nabla \cdot T+\rho b,\;\rho \dot{\varepsilon }=T\cdot D-\nabla \cdot q+\rho r.$$

Let η be the entropy density and θ the absolute temperature. According the second law of thermodynamics, we take it that the inequality

(4) $$\rho \dot{\eta }+\nabla \cdot \left(q/\theta \right)-\rho r/\theta =\sigma \geq 0$$

holds for any process compatible with the balance equations; σ is said to be the entropy production (per unit volume) [14]. Consequently, admissible constitutive equations are required to satisfy inequality (4).

Multiplying by the absolute temperature θ and substituting ∇·q−ρr from the energy Equation (3)_3_, we have

$$\rho \theta \dot{\eta }-\rho \dot{\varepsilon }+T\cdot D-\frac{1}{\theta }\;q\cdot \nabla \theta =\theta \sigma \geq 0.$$

Letting ψ=ε−θη (Helmholtz free energy density), we can write this inequality in the form

(5) $$-\rho \left(\dot{\psi }+\eta \dot{\theta }\right)+T\cdot D-\frac{1}{\theta }\;q\cdot \nabla \theta =\theta \sigma \geq 0.$$

Within the isothermal setting, the term θσ is usually referred to as the rate of (mechanical) dissipation [15]. Hence, non-dissipative models are characterized by σ=0, the vanishing of the entropy production.

The modelling of the constitutive properties is made simpler by using referential, Euclidean invariant quantities. Let

$$T_{RR}:=JF^{-1}TF^{-T},\;q_{R}:=JF^{-1}q;$$

$T_{RR}$ is called the second Piola–Kirchhoff stress. Both $T_{RR}$ and $q_{R}$ are Euclidean invariant. Under any change of frame with rotation matrix Q,

$$x\;\to \;x^{*},\;x^{*}=c\left(t\right)+Q\left(t\right)x,\;\det Q=1,$$

we have

$$F^{*}=QF,\;T_{RR}^{*}=J{\left(QF\right)}^{-1}QTQ^{T}{\left(QF\right)}^{-T}=JF^{-1}TF^{-T},\;q_{R}^{*}=J{\left(QF\right)}^{-1}Qq=JF^{-1}q.$$

The tensors C and E are invariant too in that

$$C^{*}={\left(QF\right)}^{T}\left(QF\right)=F^{T}F=C.$$

Moreover, the rate $\dot{E}$ is related to the stretching D by

(6) $$\dot{E}=F^{T}DF.$$

Now, we multiply inequality (5) by *J* (J>0); observe that Jρ is the mass density $\rho_{R}$ in the reference configuration. Accordingly, we let $\sigma_{R}=J\sigma$ and find the second-law inequality in the form

(7) $$-\rho_{R}\left(\dot{\psi }+\eta \dot{\theta }\right)+T_{RR}\cdot \dot{E}-\frac{1}{\theta }\;q_{R}\cdot \nabla_{R}\theta =\theta \sigma_{R}\geq 0.$$

If, instead, we consider the Gibbs free energy density

(8) $$\phi =\psi -\frac{1}{\rho_{R}}E\cdot T_{RR},$$

then inequality (7) takes the form

(9) $$-\rho_{R}\left(\dot{\phi }+\eta \dot{\theta }\right)-E\cdot {\dot{T}}_{RR}-\frac{1}{\theta }\;q_{R}\cdot \nabla {\;}_{R}\;\theta =\theta \sigma_{R}\geq 0.$$

In terms of the Eulerian Almansi strain,

$$E=F^{-T}E\;F^{-1}$$

the Gibbs free energy density can be expressed as

$$\phi =\psi -\frac{1}{\rho }\;E\cdot T.$$

A direct calculation shows that

(10) $${\dot{T}}_{RR}=JF^{-1}\overset{□}{\;T}F^{-T},$$

where $\overset{□}{\;T}=\dot{T}+\left(\nabla \cdot v\right)T-LT-TL^{T}$ is the Truesdell rate of T [8,9]. Accordingly, in the spatial description, inequality (9) becomes

(11) $$-\rho \left(\dot{\phi }+\eta \dot{\theta }\right)-E\;\cdot \overset{□}{\;T}-\frac{1}{\theta }\;q\cdot \nabla \theta =\theta \sigma \geq 0.$$

For later use, we observe that time differentiation of $E=F^{T}EF$ yields

(12) $$\dot{E}=F^{T}\left(\dot{E}+L^{T}E+EL\right)F=F^{T}\overset{△}{\;E}F$$

where $\overset{△}{\;E}$ denotes the Cotter–Rivlin rate [9] of E. In view of (6), it follows that

(13) $$\overset{△}{\;E}\;=D.$$

The following property [8] allows for a more general set of constitutive equations consistent with the second law; the symbol I denotes the fourth-order identity tensor.

**Lemma** **1.** *Given a second-order tensor A, let N=A/|A|, $\left|A\right|=\sqrt{A\cdot A}$. If Z is a second-order tensor such that only Z·N is known, then*

(14) Z=(Z·N)N+(I−N⊗N)G

*for any second-order tensor G. A representation formula such as (14) also holds when A, Z and G are vectors, provided that I is replaced by 1, the second-order identity tensor.*

**Proof.**  If Z is a tensor such that $Z_{\|}$ is given while $Z_{⊥}$ is undetermined, then the representation of Z allows for any tensor $Z_{⊥}$ subject to $Z_{⊥}\cdot N=0$. Now, since [(I−N⊗N)G]·N=0 for any second-order tensor G, then [(I−N⊗N)G] is any possible value of $Z_{⊥}$. Hence the relation $Z=Z_{\|}+Z_{⊥}$ results in the representation (14). □

## 3. Constitutive Relations

To describe thermo-viscoelastic effects, we let

$$\Xi_{R}:=\left(\theta ,E,T_{RR},q_{R},\dot{E},{\dot{T}}_{RR},{\dot{q}}_{R},\nabla {\;}_{R}\;\theta \right)$$

be the set of variables; in light of the model under consideration, the single variables $\dot{E}$, ${\dot{T}}_{RR}$, ${\dot{q}}_{R}$, $\nabla {\;}_{R}\;\theta$ need not be mutually independent. Hence, we let ψ,η depend on $\Xi_{R}$. In addition, we assume that η is continuous while ψ is continuously differentiable.

Upon evaluation of $\dot{\psi }$ and substitution in (7), we obtain

$$\begin{array}{cc} \rho_{R}\left(\partial_{\theta }\psi +\eta \right)\dot{\theta }+\left(\rho_{R}\partial_{E}\psi -T_{RR}\right)\cdot \dot{E}+\rho_{R}\partial_{T_{RR}}\psi \cdot {\dot{T}}_{RR}+\rho_{R}\partial_{q_{R}}\psi \cdot {\dot{q}}_{R} & \\ +\rho_{R}\partial_{\dot{E}}\psi \cdot \ddot{E}+\rho_{R}\partial_{{\dot{T}}_{RR}}\psi \cdot {\ddot{T}}_{RR}+\rho_{R}\partial_{{\dot{q}}_{R}}\psi \cdot {\ddot{q}}_{R}+\rho_{R}\partial_{\nabla {\;}_{R}\;\theta }\psi \cdot \nabla {\;}_{R}\;\dot{\theta }+\frac{1}{\theta }q_{R}\cdot \nabla {\;}_{R}\;\theta & =-\theta \sigma_{R}. \end{array}$$

The linearity and arbitrariness of $\dot{\theta }$, $\nabla {\;}_{R}\;\dot{\theta }$, $\ddot{E}$, ${\ddot{T}}_{RR}$, ${\ddot{q}}_{R}$ imply that ψ is independent of $\nabla {\;}_{R}\;\theta$, $\dot{E}$, ${\dot{T}}_{RR}$, ${\dot{q}}_{R}$ and hence

$$\psi =\psi \left(\theta ,E,T_{RR},q_{R}\right),\;\eta =-\partial_{\theta }\psi ,$$

so that the entropy inequality reduces to

(15) $$\left(\rho_{R}\partial_{E}\psi -T_{RR}\right)\cdot \dot{E}+\rho_{R}\partial_{T_{RR}}\psi \cdot {\dot{T}}_{RR}+\rho_{R}\partial_{q_{R}}\psi \cdot {\dot{q}}_{R}+\frac{q_{R}}{\theta }\cdot \nabla {\;}_{R}\;\theta =-\theta \sigma_{R}\leq 0.$$

Further restrictions depend on the arbitrariness or possible constraints on $\dot{E}$, ${\dot{T}}_{RR}$, ${\dot{q}}_{R}$, $\nabla {\;}_{R}\;\theta$. In particular, since ψ is independent of $\nabla {\;}_{R}\;\theta$, if $\dot{E}$ and ${\dot{T}}_{RR}$ are related to each other but independent of ${\dot{q}}_{R},\nabla {\;}_{R}\;\theta$, then letting ${\dot{q}}_{R}=\nabla {\;}_{R}\;\theta =0$ we write (15) in the form

(16) $$\left(\rho_{R}\partial_{E}\psi -T_{RR}\right)\cdot \dot{E}+\rho_{R}\partial_{T_{RR}}\psi \cdot {\dot{T}}_{RR}=-\theta \sigma_{R}^{ET}\leq 0,$$

where $\sigma_{R}^{ET}$ is the entropy production density $\sigma_{R}$ when ${\dot{q}}_{R}=\nabla {\;}_{R}\;\theta =0$. In terms of the Gibbs free energy density, inequality (16) becomes

(17) $$\rho_{R}\partial_{E}\phi \cdot \dot{E}+\left(\rho_{R}\partial_{T_{RR}}\phi +E\right)\cdot {\dot{T}}_{RR}=-\theta \sigma_{R}^{ET}\leq 0.$$

Likewise, if, as is usually the case, ${\dot{q}}_{R},q_{R}$ and $\nabla {\;}_{R}\;\theta$ are independent of $\dot{E},{\dot{T}}_{RR}$, then

(18) $$\rho_{R}\partial_{q_{R}}\psi \cdot {\dot{q}}_{R}+\frac{q_{R}}{\theta }\cdot \nabla {\;}_{R}\;\theta =-\theta \sigma_{R}^{Q}\leq 0.$$

Apparently, $\sigma_{R}=\sigma_{R}^{ET}+\sigma_{R}^{Q}$. Hereafter, the entropy productions $\sigma_{R}^{ET}$ and $\sigma_{R}^{Q}$ are assumed to be non-negative constitutive functions of $\Xi_{R}$ to be determined according to the constitutive model.

**Remark** **1.** *Inequality (18) is common to many approaches where both $q_{R}$ and $\nabla {\;}_{R}\;\theta$ are independent variables [16,17,18]. Since it is framed in the referential description then inequality (16) has the advantage that the material time derivative is objective [9,19,20]. Moreover, it makes consistency with thermodynamics much easier than it happens with histories [21] or summed histories [22].*

If $\dot{E}$, ${\dot{T}}_{RR}$ and ${\dot{q}}_{R}$ are independent of each other, then we have the following:

$$\partial_{T_{RR}}\psi =\partial_{q_{R}}\psi =0,\;T_{RR}=\rho_{R}\partial_{E}\psi ,\;q_{R}\cdot \nabla {\;}_{R}\;\theta =-\theta^{2}\sigma_{R}\leq 0,$$

Accordingly, ψ depends only on θ,E. In addition, $T_{RR}$ is no longer an independent variable but equals $\rho_{R}\partial_{E}\psi$, which can be viewed as the constitutive relation for $T_{RR}$, as it happens with thermo-hyperelastic materials.

It is of interest to look for the corresponding Eulerian descriptions. To this purpose, we observe that T (see (12)) and likewise q satisfy

$$\overset{□}{\;T}\;=J^{-1}F{\dot{T}}_{RR}F^{T},\;\overset{□}{\;q}\;:=\dot{q}+\left(\nabla \cdot v\right)q-Lq=J^{-1}F{\dot{q}}_{R},$$

with $\overset{□}{\;q}$ being the Truesdell rate of q.

Since $\psi =\psi (\theta ,E,T_{RR},q_{R})$, then we consider the dependence on E,T,q through $E,T_{RR},q_{R}$. The definitions of $E,T_{RR}$, and $q_{R}$ allow us to write

$$\psi =\psi \left(\theta ,E,T_{RR},q_{R}\right)=\psi \left(\theta ,E\left(E,F\right),T_{RR}\left(T,F\right),q_{R}\left(q,F\right)\right)=:\tilde{\psi }\left(\theta ,E,T,q,F\right).$$

Hence, in view of (12), we have

$$\partial_{E}\tilde{\psi }=\partial_{E}\psi \partial_{E}E,\;\partial_{E}\psi =F^{-1}\partial_{E}\tilde{\psi }F^{-T},\;\partial_{E}\psi \cdot \dot{E}=\partial_{E}\tilde{\psi }\cdot \overset{△}{\;E},\;T_{RR}\cdot \dot{E}=JT\cdot \overset{△}{\;E}$$

and, in view of (10),

$$\partial_{T_{RR}}\psi =F^{T}\partial_{T}\tilde{\psi }F,\;\partial_{T_{RR}}\psi \cdot {\dot{T}}_{RR}=\partial_{T}\tilde{\psi }\cdot \overset{□}{\;T}.$$

Likewise, we evaluate ${\dot{q}}_{R}$, $\partial_{q_{R}}\psi$ and $q_{R}\cdot \nabla {\;}_{R}\;\theta$. Consequently, inequalities (16) and (18) can be written in the Eulerian form

(19) $$\rho \partial_{T}\tilde{\psi }\cdot \overset{□}{\;T}+\left(\rho \partial_{E}\tilde{\psi }-T\right)\cdot \overset{△}{\;E}\;=-\theta \sigma^{ET}\leq 0,\;\rho \partial_{q}\tilde{\psi }\cdot \overset{□}{\;q}+\frac{q}{\theta }\cdot \nabla \theta =-\theta \sigma^{Q}\leq 0.$$

where $\sigma^{ET}$ and $\sigma^{Q}$ are non-negative constitutive functions of Ξ and $\sigma^{ET}+\sigma^{Q}=\sigma$.

In terms of the Gibbs free energy $\phi =\tilde{\phi }\left(\theta ,E,T,q\right)$, inequality (17) can be given the form

(20) $$\rho \partial_{E}\tilde{\phi }\;\cdot \overset{△}{\;E}+\left(\rho \partial_{T}\tilde{\phi }+E\right)\cdot \overset{□}{\;T}=-\theta \sigma^{ET}\leq 0.$$

It is worth remarking that the application of (5) in connection with $\tilde{\psi }\left(\theta ,E,T,q,F\right)$ would result in inequalities different from (19) though the entropy production is invariant, namely

$$J\sigma =\sigma_{R}^{ET}+\sigma_{R}^{Q}.$$

## 4. Hypo-Thermoelastic Solids

Assume that $\sigma_{R}^{ET}=0$, $\sigma_{R}^{Q}=0$ and

$$\partial_{T_{RR}}\psi \neq 0,\;\partial_{q_{R}}\psi \neq 0.$$

Hence, Equations (16) and (18) become

(21) $$\rho_{R}\partial_{T_{RR}}\psi \cdot {\dot{T}}_{RR}=\left(T_{RR}-\rho_{R}\partial_{E}\psi \right)\cdot \dot{E},$$

(22) $$\rho_{R}\partial_{q_{R}}\psi \cdot {\dot{q}}_{R}=-\frac{1}{\theta }q_{R}\cdot \nabla {\;}_{R}\;\theta .$$

Equations (21) and (22) are said to describe hypo-thermoelastic solids; owing to the condition $\sigma_{R}=0$, such models are viewed as non-dissipative. More explicit relations for ${\dot{T}}_{RR}$ and ${\dot{q}}_{R}$ follow by appealing to Lemma 1.

First, let $N=\partial_{T_{RR}}\psi /\left|\partial_{T_{RR}}\psi \right|$ and $Z=\dot{T}RR$. The representation formula (14) is applicable in that Z·N is known,

$$Z\cdot N=\frac{\left(T_{RR}-\rho_{R}\partial_{E}\psi \right)\cdot \dot{E}}{\rho_{R}\left|\partial_{T_{RR}}\psi \right|}.$$

Accordingly, we select $G=J_{RR}\dot{E}$, where $J_{RR}$ is an arbitrary fourth-order tensor, and let $J_{RR}$ depend on $\theta ,E,T_{RR},q_{R}$. It follows from (21) that

$${\dot{T}}_{RR}=\left[\frac{\left(T_{RR}-\rho_{R}\partial_{E}\psi \right)\cdot \dot{E}}{\rho_{R}{\left|\partial_{T_{RR}}\psi \right|}^{2}}\right]\partial_{T_{RR}}\psi +\left(I-N⊗N\right)J_{RR}\dot{E}.$$

This equation can be written in a compact form by letting

(23) $$\begin{array}{cc} C_{RR} & =\frac{1}{\rho_{R}\left|\partial_{T_{RR}}\psi \right|}N⊗\left(T_{RR}-\rho_{R}\partial_{E}\psi \right)+\left(I-N⊗N\right)J_{RR}\\ & =J_{RR}+\frac{1}{\rho_{R}{\left|\partial_{T_{RR}}\psi \right|}^{2}}\partial_{T_{RR}}\psi ⊗\left(T_{RR}-\rho_{R}\partial_{E}\psi -\rho_{R}J_{RR}^{T}\partial_{T_{RR}}\psi \right). \end{array}$$

We can then write (21) in the form

(24) $${\dot{T}}_{RR}=C_{RR}\left(\theta ,E,T_{RR},q_{R}\right)\dot{E}.$$

Likewise, we can also apply (14) with the vectors $N=\partial_{q_{R}}\psi /\left|\partial_{q_{R}}\psi \right|$, $Z={\dot{q}}_{R}$ and $G=J_{R}\nabla {\;}_{R}\;\theta$, where $J_{R}$ is is an arbitrary second-order tensor. Using (22), we have

$$Z\cdot N=-\frac{q_{R}\cdot \nabla {\;}_{R}\;\theta }{\rho_{R}\theta \left|\partial_{q_{R}}\psi \right|}.$$

and then

$${\dot{q}}_{R}=-\left[\frac{q_{R}\cdot \nabla {\;}_{R}\;\theta }{\rho_{R}\theta {\left|\partial_{q_{R}}\psi \right|}^{2}}\right]\partial_{q_{R}}\psi +\left(1-N⊗N\right)J_{R}\nabla {\;}_{R}\;\theta ,$$

where $J_{R}=J_{R}\left(\theta ,E,T_{RR},q_{R}\right)$. Hence, letting

(25) $$\begin{array}{cc} K_{R} & =\frac{1}{\rho_{R}\theta \left|\partial_{q_{R}}\psi \right|}N⊗q_{R}-\left(1-N⊗N\right)J_{R}\\ & =J_{R}+\frac{1}{\rho_{R}{\left|\partial_{q_{R}}\psi \right|}^{2}}\partial_{q_{R}}\psi ⊗\left(q_{R}/\theta +\rho_{R}J_{R}^{T}\partial_{q_{R}}\psi \right), \end{array}$$

we can write (22) in the compact form,

(26) $${\dot{q}}_{R}=-K_{R}\left(\theta ,E,T_{RR},q_{R}\right)\nabla {\;}_{R}\;\theta .$$

Relations (24) and (26) describe the constitutive properties of hypo-thermoelastic solids and $C_{RR}$ and $K_{R}$ are referred to as hypo-elastic and hypo-thermal tensors, respectively. By the arbitrariness of $J_{RR}$ and $J_{R}$, it follows that there are infinitely many tensors $C_{RR}$ and $K_{R}$ compatible with a given free energy ψ. Moreover, $C_{RR}$ and $K_{R}$ need not be positive definite and this is a remarkable difference from the positive definiteness of the elastic tensor and the conductivity tensor.

**Remark** **2.** *Relative to rigid heat conductors, if we assume $\varepsilon =c_{v}\theta +\varepsilon_{0}$, $c_{v}>0$, the balance of internal energy takes the form,*

$$\rho_{R}c_{v}\dot{\theta }=-\nabla {\;}_{R}\;\cdot q_{R}+\rho_{R}r$$

*and hence, it follows from (26) that*

$$\rho_{R}c_{v}\ddot{\theta }=\nabla {\;}_{R}\;\cdot \left(K_{R}\nabla {\;}_{R}\;\theta \right)+\rho_{R}\dot{r}.$$

*This equation is the same as that resulting from Green and Naghdi’s type II theory (see [23,24,25,26]). It is hyperbolic if $K_{R}$ is positive-definite, but this is not implied by thermodynamics.*

Otherwise, if hypo-thermoelastic constitutive relations are given in advance, for instance

(27) $${\dot{T}}_{RR}={\hat{C}}_{RR}\dot{E},\;{\dot{q}}_{R}={\hat{K}}_{R}\nabla {\;}_{R}\;\theta ,$$

then from (21) and (22), we obtain

$$\begin{array}{cc} \rho_{R}\partial_{T_{RR}}\psi \cdot {\hat{C}}_{RR}\dot{E} & =\left(T_{RR}-\rho_{R}\partial_{E}\psi \right)\cdot \dot{E},\\ \rho_{R}\partial_{q_{R}}\psi \cdot {\hat{K}}_{R}\nabla {\;}_{R}\;\theta & =-\frac{q_{R}}{\theta }\cdot \nabla {\;}_{R}\;\theta . \end{array}$$

and the arbitrariness of $\dot{E}$ and $\nabla {\;}_{R}\;\theta$ implies

(28) $$T_{RR}-\rho_{R}\partial_{E}\psi =\rho_{R}{\hat{C}}_{RR}^{T}\partial_{T_{RR}}\psi ,\;q_{R}=-\rho_{R}\theta {\hat{K}}_{R}^{T}\partial_{q_{R}}\psi .$$

In [27], the existence of a thermodynamic potential ψ satisfying (21) or (22) is investigated by exploiting the overdetermined systems (28) with assigned tensors ${\hat{C}}_{RR}$ and ${\hat{K}}_{R}$.

**Proposition** **1.** *If we replace (28) into (23) and (25), we obtain*

$$\begin{array}{cc} C_{RR} & =J_{RR}+\frac{1}{|\partial_{T_{RR}}{\psi |}^{2}}\partial_{T_{RR}}\psi ⊗\left[\left({\hat{C}}_{RR}^{T}-J_{RR}^{T}\right)\partial_{T_{RR}}\psi \right],\\ K_{R} & =J_{R}+\frac{1}{|\partial_{q_{R}}{\psi |}^{2}}\partial_{q_{R}}\psi ⊗\left[\left({\hat{K}}_{R}^{T}-J_{R}^{T}\right)\partial_{q_{R}}\psi \right]. \end{array}$$

*Hence, by properly choosing the arbitrary terms (namely, if we let $J_{RR}={\hat{C}}_{RR}$ and $J_{R}={\hat{K}}_{R}$), we obtain the expected identities $C_{RR}={\hat{C}}_{RR}$ and $K_{R}={\hat{K}}_{R}$.*

A similar approach can be developed if the Gibbs free energy ϕ is given instead of ψ. From (17) with $\sigma_{R}^{ET}=0$, we have

(29) $$\rho_{R}\partial_{E}\phi \cdot \dot{E}+\left(\rho_{R}\partial_{T_{RR}}\phi +E\right)\cdot {\dot{T}}_{RR}=0,$$

and assuming $\partial_{E}\phi \neq 0$, it follows

$$\dot{E}=-\left[\frac{\left(E+\rho_{R}\partial_{T_{RR}}\psi \right)\cdot {\dot{T}}_{RR}}{\rho_{R}{\left|\partial_{E}\phi \right|}^{2}}\right]\partial_{E}\phi .$$

Hence, by paralleling previous arguments, we obtain

(30) $$\dot{E}=H_{RR}{\dot{T}}_{RR},$$

where

(31) $$H_{RR}=X_{RR}-\frac{1}{\rho_{R}{\left|\partial_{E}\phi \right|}^{2}}\partial_{E}\phi ⊗\left(E+\rho_{R}\partial_{T_{RR}}\phi +\rho_{R}X_{RR}^{T}\partial_{E}\phi \right).$$

with $X_{RR}$ being an arbitrary (possibly vanishing) fourth-order tensor. Two further examples are now given by starting with the potential, ϕ or ψ.

**Example** **1.** *Let ϕ be the Gibbs free energy defined by (8), and let*

$$\rho_{R}\psi \left(\theta ,T_{RR}\right)=\rho_{R}\psi_{0}\left(\theta \right)+\frac{1}{2}\nu \left(\theta \right){\left|T_{RR}\right|}^{2}+\frac{1}{2}\int_{0}^{|T_{RR}{|}^{2}}u\beta \left(\theta ,u\right)\;du,$$

*with α and β being real-valued functions. Consequently,*

$$\rho_{R}\partial_{T_{RR}}\phi =\left[\nu \left(\theta \right)+|T_{RR}{|}^{2}\beta (\theta ,|T_{RR}{|}^{2})\right]T_{RR}-E,\;\rho_{R}\partial_{E}\phi =-T_{R^{R}},$$

*whence*

$$E+\rho_{R}\partial_{T_{RR}}\phi =-[\nu \left(\theta \right)+|T_{RR}{|}^{2}\beta (\theta ,|T_{RR}{|}^{2})]\rho_{R}\partial_{E}\phi .$$

*Let $X_{RR}=\mu I$. From (31), we have the following:*

$$H_{RR}\left(\theta ,E\right)=\mu I+\beta (\theta ,|T_{RR}{|}^{2})T_{RR}⊗T_{RR}+\left(\nu -\mu \right)\frac{T_{RR}}{|T_{RR}|}⊗\frac{T_{RR}}{|T_{RR}|}.$$

*Apparently, $H_{RR}$ enjoys the major symmetry. Choosing μ=α, we obtain*

$$H_{RR}\left(\theta ,E\right)=\nu \left(\theta \right)I+\beta (\theta ,|T_{RR}{|}^{2})T_{RR}⊗T_{RR}.$$

*The resulting constitutive equation in the form (30) is generated by the well known Prandtl–Reuss work hardening plasticity theory provided that E and $T_{RR}$ are replaced by their deviatoric parts (see [4] Sect. C).*

**Example** **2.** *Let M be a non-singular, fully symmetric, fourth-order tensor; M be a non-singular, symmetric, second-order tensor; and G be a smooth function from Sym to Sym. Consider the free energy ψ defined by*

(32) $$\rho_{R}\;\psi =\rho_{R}\;\psi_{0}\left(\theta \right)+\int_{0}^{E}G\left(u\right)\cdot du+\frac{1}{2}\left[T_{RR}-G\left(E\right)\right]\cdot M\left(\theta \right)\left[T_{RR}-G\left(E\right)\right]+\frac{1}{2}q_{R}\cdot M\left(\theta \right)q_{R},$$

*where u∈ Sym. Observe that $\rho_{R}\partial_{q_{R}}\psi =M\left(\theta \right)q_{R}$ and*

$$\rho_{R}\partial_{T_{RR}}\psi =M\left(\theta \right)\left[T_{RR}-G\left(E\right)\right],\;\rho_{R}\partial_{E}\psi =G\left(E\right)-{\left[G^{\prime }\left(E\right)\right]}^{T}M\left(\theta \right)\left[T_{RR}-G\left(E\right)\right].$$

*As a consequence, we infer that (28) holds with*

$${\hat{C}}_{RR}^{T}=M^{-1}\left(\theta \right)+{\left[G^{\prime }\left(E\right)\right]}^{T},\;{\hat{K}}_{R}^{T}=-\frac{1}{\theta }M^{-1}\left(\theta \right)$$

*Then, in light of Remark 3, we let $J_{RR}={\hat{C}}_{RR}$ and $J_{R}={\hat{K}}_{R}$ into (23) and (25) to obtain*

(33) $${\dot{T}}_{RR}=\left[G^{\prime }\left(E\right)+M^{-1}\left(\theta \right)\right]\dot{E},\;{\dot{q}}_{R}=-\frac{1}{\theta }M^{-1}\left(\theta \right)\nabla {\;}_{R}\;\theta .$$

*In the linear case, G(E)=LE, L being a (not necessarily symmetrical) fourth-order tensor, we have the following:*

$${\dot{T}}_{RR}=C_{RR}\;\dot{E},\;C_{RR}:=L+M^{-1}.$$

## 5. Hypo-Elastic Models with Thermal Dissipation

Unlike hypo-thermoelastic models, which exhibit no entropy production, Maxwell–Cattaneo and Fourier-like hypoelastic models allow for a non-negative value of $\sigma_{R}$. A quite general class of hypo-elastic models with thermal dissipation is characterized by $\sigma_{R}^{ET}=0$ but $\sigma_{R}\equiv \sigma_{R}^{Q}\geq 0$, so that (21) still holds along with (18). As in the previous section, the assumption $\partial_{T_{RR}}\psi \neq 0$ leads to (24), whereas (18) can be satisfied in two different ways, depending on whether it is $\partial_{q_{R}}\psi =0$ or $\partial_{q_{R}}\psi \neq 0$.

### 5.1. Fourier-Like Models

Assuming $\partial_{q_{R}}\psi =0$, it follows that $\psi =\psi (\theta ,E,T_{RR})$ and (18) reduces to the well-known Fourier inequality

(34) $$q_{R}\cdot \nabla {\;}_{R}\;\theta =-\theta^{2}\sigma_{R}^{Q}\leq 0.$$

This condition is satisfied, for example, by letting $q_{R}$ be given by Fourier’s law, $q_{R}=-\kappa \nabla {\;}_{R}\;\theta$, where κ is a positive-definite second-order tensor. In that case, we have $\theta^{2}\sigma_{R}^{Q}=\nabla {\;}_{R}\;\theta \cdot \kappa \nabla {\;}_{R}\;\theta$.

The same result follows also by using the representation formula (14). Let A be any symmetric, non-singular, second-order tensor, possibly parameterized by the temperature θ. Assuming $A\nabla {\;}_{R}\;\theta \neq 0$ and applying (14) with $N=A\nabla {\;}_{R}\;\theta /\left|A\nabla {\;}_{R}\;\theta \right|$, $Z=A^{-1}q_{R}$, and G=0, it follows from (34) that

$$q_{R}=-\theta^{2}\sigma_{R}^{Q}A^{2}\nabla {\;}_{R}\;\theta /{\left|A\nabla {\;}_{R}\;\theta \right|}^{2}.$$

Hence, letting $\kappa =A^{2}$ and $\sigma_{R}^{Q}=\nabla {\;}_{R}\;\theta \cdot \kappa \nabla {\;}_{R}\;\theta /\theta^{2}$, we recover Fourier’s law $q_{R}=-\kappa \nabla {\;}_{R}\;\theta$.

### 5.2. Maxwell–Cattaneo-like Models

Assume $\partial_{q_{R}}\psi \neq 0$. Equation (18) can then be written in the form

$$\frac{\partial_{q_{R}}\psi }{|\partial_{q_{R}}\psi |}\cdot {\dot{q}}_{R}=-\frac{1}{\rho_{R}\left|\partial_{q_{R}}\psi \right|}\left(\frac{q_{R}}{\theta }\cdot \nabla {\;}_{R}\;\theta +\theta \sigma_{R}^{Q}\right).$$

Applying (14) with $N=\partial_{q_{R}}\psi /\left|\partial_{q_{R}}\psi \right|$, $Z={\dot{q}}_{R}$ and $G=J_{R}\nabla {\;}_{R}\;\theta$, we obtain

(35) $${\dot{q}}_{R}=-K_{R}\nabla {\;}_{R}\;\theta -\sigma_{R}^{Q}Q_{R},$$

where $K_{R}=K_{R}\left(\theta ,E,T_{RR},q_{R}\right)$ is given by (25) and

$$Q_{R}=\frac{\theta \;\partial_{q_{R}}\psi }{\rho_{R}{\left|\partial_{q_{R}}\psi \right|}^{2}}.$$

For definiteness, we now show that a class of models for heat conduction, of the Maxwell–Cattaneo type [28], follows from (35). Let ψ depend on $q_{R}$ via $\xi =|q_{R}{|}^{n}$, n≥2. Hence,

$$\partial_{q_{R}}\psi =n\;\partial_{\xi }\psi \;{\left|q_{R}\right|}^{n-2}\;q_{R},\;\partial_{\xi }\psi \neq 0$$

and inequality (18) becomes

$$\left(n\rho_{R}\partial_{\xi }\psi {\left|q_{R}\right|}^{n-2}{\dot{q}}_{R}+\frac{1}{\theta }\nabla {\;}_{R}\;\theta \right)\cdot q_{R}=-\theta \sigma_{R}^{Q}\leq 0.$$

Let $\sigma_{R}^{Q}={\left|q_{R}\right|}^{2}/\theta \kappa$, where κ is a positive-valued function. In view of (35), we have the following:

(36) $$n\rho_{R}\partial_{\xi }\psi {\left|q_{R}\right|}^{n-2}{\dot{q}}_{R}+\frac{1}{\theta }\nabla {\;}_{R}\;\theta =-\frac{1}{\kappa }q_{R}.$$

Consequently,

$$\kappa n\rho_{R}\theta \partial_{\xi }\psi {\left|q_{R}\right|}^{n-2}{\dot{q}}_{R}+q_{R}=-\kappa \nabla {\;}_{R}\;\theta$$

can be viewed as a Maxwell–Cattaneo equation with

$$\tau =\kappa n\rho_{R}\theta \partial_{\xi }\psi {\left|q_{R}\right|}^{n-2}$$

playing the role of relaxation time and κ representing the heat conductivity. If n=2, then

$$\tau =2\kappa \rho_{R}\theta \partial_{\xi }\psi .$$

In this case, τ reduces to a function of the temperature alone when κ=κ(θ) and ψ is a quadratic function of $q_{R}$.

## 6. Thermo-Viscoelastic and Viscoplastic Models

Models of thermo-viscoelastic materials are characterized by non-negative entropy productions $\sigma_{R}^{ET}$ and $\sigma_{R}^{Q}$ independent of $\dot{E}$, ${\dot{T}}_{RR}$, ${\dot{q}}_{R}$ and $\nabla {\;}_{R}\;\theta$. In general, both the free energy and the entropy productions are functions of $\left(\theta ,E,T_{RR},q_{R}\right)$. We recognize two different classes of models.

Assuming $\partial_{T_{RR}}\psi ,\partial_{q_{R}}\psi \neq 0$ and applying the representation formula (14), we can rewrite (16) and (18) in the form

(37) $${\dot{T}}_{RR}=C_{RR}\dot{E}-P_{RR}+\left(I-N⊗N\right)G_{RR},\;{\dot{q}}_{R}=-K_{R}\nabla {\;}_{R}\;\theta -Q_{R}+\left(I-n⊗n\right)g_{R},$$

where

$$N=\frac{\partial_{T_{RR}}\psi }{|\partial_{T_{RR}}\psi |},\;C_{RR}=N⊗\frac{T_{RR}-\rho_{R}\partial_{E}\psi }{\rho_{R}\left|\partial_{T_{RR}}\psi \right|},\;P_{RR}=\frac{\theta \sigma_{R}^{ET}N}{\rho_{R}\left|\partial_{T_{RR}}\psi \right|},$$

$$n=\frac{\partial_{q_{R}}\psi }{|\partial_{q_{R}}\psi |},\;K_{R}=n⊗\frac{q_{R}}{\rho_{R}\theta \left|\partial_{q_{R}}\psi \right|},\;Q_{R}=\frac{\theta \sigma_{R}^{Q}n}{\rho_{R}\left|\partial_{q_{R}}\psi \right|},$$

$G_{RR}$ and $g_{R}$ being respectively an arbitrary tensor and an arbitrary vector.

In terms of the free enthalpy ϕ, Equation (37)_1_ takes the form

(38) $$\dot{E}=H_{RR}{\dot{T}}_{RR}-S_{RR}+\left(I-U⊗U\right)G_{RR},$$

where

$$U=\frac{\partial_{E}\phi }{|\partial_{E}\phi |},\;H_{RR}=-U⊗\frac{E+\rho_{R}\partial_{T_{RR}}\phi }{\rho_{R}\left|\partial_{E}\phi \right|},\;S_{RR}=\frac{\theta \sigma_{R}^{ET}U}{\rho_{R}\left|\partial_{E}\phi \right|}.$$

Incremental relations of this type describe a thermo-viscoelastic behaviour when $\sigma_{R}^{ET}$ is smooth. If instead $\sigma_{R}^{ET}$ is only piecewise smooth and vanishes in a suitable open region then a rate-dependent thermo-viscoplastic behaviour is concerned; for the one-dimensional isothermal case, see for instance [8,29,30].

Otherwise, when $\partial_{T_{RR}}\psi =\partial_{q_{R}}\psi =0$ relations (16) and (18) reduce to

$$\left(T_{RR}-\rho_{R}\partial_{E}\psi \right)\cdot \dot{E}=\theta \sigma_{R}^{ET},\;q_{R}\cdot \nabla {\;}_{R}\;\theta =-\theta^{2}\sigma_{R}^{Q}.$$

The latter equation has been scrutinized in Section 5.1. Concerning $T_{RR}$, let M be any symmetric, non-singular fourth-order tensor, possibly parameterized by θ. Assuming $M\dot{E}\neq 0$ and applying (14) with $N=M\dot{E}/\left|M\dot{E}\right|$, $Z=M^{-1}\left[T_{RR}-\rho_{R}\partial_{E}\psi \right]$ and G=0, we can express $T_{RR}$ in the form

(39) $$T_{RR}=\rho_{R}\partial_{E}\psi +\frac{\theta \sigma_{R}^{ET}}{|M\dot{E}{|}^{2}}M^{2}\dot{E}.$$

We now describe some simple examples of (37)–(39) via special choices of the free energy and the non-negative functions $\sigma_{R}^{ET}$ and $\sigma_{R}^{Q}$.

### 6.1. Thermo-Viscoelastic Behaviour

Let M be a fully symmetric, positive-definite, fourth-order tensor; M be a symmetric, positive-definite, second-order tensor; G be a smooth function from Sym to Sym; and ψ be given by (32) as in Example 2. Then, we have

$$\rho_{R}\partial_{T_{RR}}\psi =M\left[T_{RR}-G\left(E\right)\right],\;\;\rho_{R}\partial_{E}\psi =G\left(E\right)+{\left[G^{\prime }\left(E\right)\right]}^{T}M\left[T_{RR}-G\left(E\right)\right],\;\;\rho_{R}\partial_{q_{R}}\psi =Mq_{R},$$

and we can apply (37), where

$$N=\frac{M[T_{RR}-G\left(E\right)]}{\left|M[T_{RR}-G\left(E\right)]\right|},\;n=\frac{Mq_{R}}{|Mq_{R}|}.$$

Since [X⊗Y]Z=(Y·Z)X for tensors or vectors X,Y,Z, we have

$$C_{RR}\dot{E}=\left[N⊗\left(M^{-1}+{\left[G^{\prime }\left(E\right)\right]}^{T}\right)N\right]\dot{E}=\left[N\cdot \left(M^{-1}+G^{\prime }\left(E\right)\right)\dot{E}\right]N,$$

$$K_{R}\nabla {\;}_{R}\;\theta =\frac{1}{\theta }\left[n⊗M^{-1}n\right]\nabla {\;}_{R}\;\theta =\frac{1}{\theta }\left(n\cdot M^{-1}\nabla {\;}_{R}\;\theta \right)n$$

Now, letting

$$\sigma_{R}^{ET}=\frac{1}{\theta \tau_{T}}\left[T_{RR}-G\left(E\right)\right]\cdot M\left[T_{RR}-G\left(E\right)\right],\;\sigma_{R}^{Q}=\frac{1}{\theta \tau_{q}}q_{R}\cdot Mq_{R},\;\tau_{T},\tau_{q}>0,$$

from (37), we have the following:

$${\dot{T}}_{RR}=\left[N\cdot \left([M^{-1}+G^{\prime }\left(E\right)\right]\dot{E}-\frac{1}{\tau_{T}}\left[T_{RR}-G\left(E\right)\right]\right)]N+\left(I-N⊗N\right)G_{RR},$$

$${\dot{q}}_{R}=-\left[n\cdot \left(\frac{1}{\theta }M^{-1}\nabla {\;}_{R}\;\theta +\frac{1}{\tau_{q}}q_{R}\right)\right]n+\left(1-n⊗n\right)g_{R},$$

Finally, we obtain

(40) $${\dot{T}}_{RR}=\left[M^{-1}+G^{\prime }\left(E\right)\right]\dot{E}-\frac{1}{\tau_{T}}\left[T_{RR}-G\left(E\right)\right],\;{\dot{q}}_{R}=-\frac{1}{\theta }M^{-1}\nabla {\;}_{R}\;\theta -\frac{1}{\tau_{q}}q_{R},$$

simply by choosing $G_{RR}$ and $g_{R}$ equal to the right-hand members of the equalities in (40).

When G is linear, namely $G\left(E\right)=G_{\infty }E$, from (40), we recover the standard linear solid (or Zener) model with Mawell–Cattaneo heat conduction,

(41) $${\dot{T}}_{RR}+\frac{1}{\tau_{T}}\left(T_{RR}-G_{\infty }E\right)=G_{0}\dot{E},\;{\dot{q}}_{R}+\frac{1}{\tau_{q}}q_{R}=-\kappa \nabla {\;}_{R}\;\theta$$

where $G_{0}=G_{\infty }+M^{-1}$ and $\kappa =M^{-1}/\theta$. Here, $G_{0}$ and $G_{\infty }$ stand for the usual elastic and relaxation moduli, respectively, whereas κ stands for the conductivity tensor. The corresponding free energy is

$$\rho_{R}\psi =\rho_{R}\psi_{0}\left(\theta \right)+\frac{1}{2}E\cdot G_{\infty }E+\frac{1}{2}\left[T_{RR}-G_{\infty }E\right]\cdot {\left(G_{0}-G_{\infty }\right)}^{-1}\left[T_{RR}-G_{\infty }E\right]+\frac{1}{2\theta }q_{R}\cdot \kappa^{-1}q_{R}.$$

For solids, $G_{\infty }>0$. In the special case $G_{\infty }=0$, we obtain the Maxwell model for fluids.

$${\dot{T}}_{RR}+\frac{1}{\tau_{T}}T_{RR}=G_{0}\dot{E},\;{\dot{q}}_{R}+\frac{1}{\tau_{q}}q_{R}=-\kappa \nabla {\;}_{R}\;\theta .$$

It is worth looking for the possible relation between (41)_1_ and the classical linear equation in Equation (1). Under the small strain assumption, we observe that

$$E≃\varepsilon ,\;T_{RR}≃T,$$

the approximation being motivated by linearity. Hence, we might replace (1) with

$$T_{RR}\left(t\right)=G_{0}E\left(t\right)+\int_{0}^{\infty }G^{\prime }\left(s\right)E\left(t-s\right)ds.$$

As an example, let

$$G\left(s\right):=G_{\infty }+\left(G_{0}-G_{\infty }\right)\exp\left(-s/\tau \right),\;\tau >0.$$

Hence, upon time differentiation, we find

$${\dot{T}}_{RR}\left(t\right)=G_{0}\dot{E}\left(t\right)-\frac{1}{\tau }\left(G_{0}-G_{\infty }\right)\int_{0}^{\infty }\exp\left(-s/\tau \right)\dot{E}\left(t-s\right)ds,$$

whence (41)_1_ follows.

### 6.2. The Bingham–Norton Model

This model describes the behaviour of an elastic-perfectly viscoplastic solid. In our setting, it can be derived by assuming $\partial_{E}\psi =\partial_{q_{R}}\psi =0$. Accordingly, $\rho_{R}\partial_{E}\phi =-T_{RR}$, and then from (38), we obtain

(42) $$\dot{E}=H_{RR}{\dot{T}}_{RR}+\sigma_{R}^{ET}\theta \frac{T_{RR}}{|T_{RR}{|}^{2}},$$

where (for simplicity, we let $G_{RR}=0$)

$$H_{RR}\left(\theta ,T_{RR}\right)=\frac{\rho_{R}}{|T_{RR}{|}^{2}}T_{RR}⊗\partial_{T_{RR}}\psi .$$

Choosing $\lambda ,S_{y}>0$ and letting

(43) $$\sigma_{R}^{ET}=\left\{\begin{array}{cc} 0 & \;\mathrm{if}\;|T_{RR}|\leq S_{y},\\ \frac{1}{\theta \lambda^{n}}{\left|T_{RR}\right|}^{n}\left(|T_{RR}|-S_{y}\right) & \mathrm{otherwise}, \end{array}\right.,$$

we recover the Bingham–Norton model [31] for n>1. If, instead, n=1 and $S_{y}=0$, the model reduces to

$$\dot{E}=H_{RR}{\dot{T}}_{RR}+\frac{1}{\lambda }T_{RR},$$

which may be viewed as a generalized Maxwell–Wiechert model. In any case, the free energy is a generic function $\psi =\psi (\theta ,T_{RR})$.

The Bingham–Norton model (42) and (43) allows for a special decomposition of the deformation rate, $\dot{E}={\dot{E}}^{e}+{\dot{E}}^{vp}$. The elastic strain rate is represented by

$${\dot{E}}^{e}=H_{RR}{\dot{T}}_{RR},$$

whereas the rate of visco-plastic strain is only a function of the stress and depends on the initial yield stress $S_{y}$ (there is no influence of hardening)

$${\dot{E}}^{vp}=\left\{\begin{array}{cc} 0 & \;\mathrm{if}\;|T_{RR}|\leq S_{y},\\ \frac{1}{\lambda^{n}}{\left|T_{RR}\right|}^{n-2}\left(|T_{RR}|-S_{y}\right)T_{RR} & \mathrm{otherwise}. \end{array}\right.$$

This happens by letting

$$\sigma_{R}^{ET}=\frac{1}{\theta }\frac{|T_{RR}{|}^{n}}{\lambda^{n}}\left(\right|T_{RR}|-S_{y})H(S_{y}-|T_{RR}\left|\right),$$

where *H* denotes the Heaviside step function.

### 6.3. The Kelvin–Voigt Model

Let $\rho_{R}\psi =\rho_{R}\psi_{0}\left(\theta \right)+\frac{1}{2}G_{0}E\cdot E$ and $\sigma_{R}^{ET}=\theta^{-1}{\left|M\dot{E}\right|}^{2}$. Then, (39) becomes

(44) $$T_{RR}=G_{0}E+M^{2}\dot{E}.$$

When large deformations are involved, this is the three-dimensional continuum-mechanics version of the well-known rheological model realized by a spring and a dashpot in parallel, where the stress is the sum of terms proportional to the strain and the strain rate. Under the small strain assumption, the linear constitutive Equation (44) is approximated by replacing E and $\dot{E}$ with ε and $\dot{\varepsilon }$, respectively.

## 7. Thermo-Viscoelastic and Viscoplastic Models in the Spatial Description

The difficulty in formulating incremental, or rate-type, models in the spatial description of finite deformations is related to the appropriate selection of objective time derivatives. This difficulty is overcome here by translating the visco-thermoelastic models previously formulated by means of (19) in the spatial description.

Taking into account that $\overset{△}{\;E}\;=D$, Equation (19) are written in the form

(45) $$\rho \partial_{T}\tilde{\psi }\cdot \overset{□}{\;T}+\left(\rho \partial_{E}\tilde{\psi }-T\right)\cdot D\;=-\theta \sigma^{ET},\;\rho \partial_{q}\tilde{\psi }\cdot \overset{□}{\;q}+\frac{q}{\theta }\cdot \nabla \theta =-\theta \sigma^{Q}.$$

where both the free energy and the non-negative entropy productions are functions of (θ,E,T,q). Assuming $\partial_{T}\psi ,\partial_{q}\psi \neq 0$ are nonzero and applying the same arguments of the previous sections, we can rewrite (45) in the form

(46) $$\overset{□}{\;T}=C\dot{E}-P+\left(I-N⊗N\right)G,\;\overset{□}{\;q}=-K\nabla \theta -Q+\left(I-n⊗n\right)g,$$

where

$$N=\frac{\partial_{T}\psi }{|\partial_{T}\psi |},\;C=N⊗\frac{T-\rho \partial_{E}\psi }{\rho |\partial_{T}\psi |},\;P=\frac{\theta \sigma^{ET}N}{\rho |\partial_{T}\psi |},$$

$$n=\frac{\partial_{q}\psi }{|\partial_{q}\psi |},\;K=n⊗\frac{q}{\rho \theta |\partial_{q}\psi |},\;Q=\frac{\theta \sigma^{Q}n}{\rho |\partial_{q}\psi |},$$

with G and g being, respectively, an arbitrary tensor and an arbitrary vector.

In terms of the free enthalpy ϕ, Equation (38) takes the form

(47) $$D=H\overset{□}{\;T}-S+(I-U⊗U)G,$$

where

$$U=\frac{\partial_{E}\phi }{|\partial_{E}\phi |},\;H=-U⊗\frac{E+\rho \partial_{T}\phi }{\rho |\partial_{E}\phi |},\;S=\frac{\theta \sigma^{ET}U}{\rho |\partial_{E}\phi |}.$$

G being an arbitrary second-order tensor.

### 7.1. Thermo-Viscoelastic Behaviour

Let A be a fully symmetric, positive-definite, fourth-order tensor and A be a symmetric, positive-definite, second-order tensor. Optionally, both A and A are parameterized by θ. Moreover, let F be a monotone function from Sym to Sym. Given

$$\sigma^{ET}=\frac{1}{\theta \tau_{T}}\left[T-F\left(E\right)\right]\cdot A\left[T-F\left(E\right)\right],\;\sigma^{Q}=\frac{1}{\theta \tau_{q}}q\cdot Aq,\;\tau_{T},\tau_{q}>0.$$

both are non-negative, and from (45), we have the following:

(48) $$\begin{array}{cc} \left[\rho \partial_{E}\psi -T\right]\cdot D+\rho \partial_{T}\psi \cdot \overset{□}{\;T} & =-\frac{1}{\tau_{T}}\left(T-F\left(E\right)\right)\cdot A\left(T-F\left(E\right)\right),\\ \rho \partial_{q}\tilde{\psi }\cdot \overset{□}{\;q}+\frac{q}{\theta }\cdot \nabla \theta & =-\frac{1}{\tau_{q}}q\cdot Aq. \end{array}$$

Now, letting ψ such that

$$\rho \partial_{T}\psi =A\left[T-F\left(E\right)\right],\;\rho \partial_{E}\psi =F\left(E\right)-{\left[F^{\prime }\left(E\right)\right]}^{T}A\left[T-F\left(E\right)\right],\;\rho \partial_{q}\psi =Aq,$$

we obtain

$$\begin{array}{cc} A\left[T-F\left(E\right)\right]\cdot \left[\overset{□}{\;T}-\left(A^{-1}+F^{\prime }\left(E\right)\right)D\right] & =-\frac{1}{\tau_{T}}\left[T-F\left(E\right)\right]\cdot A\left[T-F\left(E\right)\right],\\ Aq\cdot \overset{□}{\;q}+\frac{q}{\theta }\cdot \nabla \theta & =-\frac{1}{\tau_{q}}q\cdot Aq. \end{array}$$

These equations are certainly satisfied if

(49) $$\overset{□}{\;T}-\left[A^{-1}+F^{\prime }\left(E\right)\right]D+\frac{1}{\tau_{T}}\left(T-F\left(E\right)\right)=0,\;\overset{□}{\;q}+\frac{1}{\theta }A^{-1}\nabla \theta +\frac{q}{\tau_{q}}=0.$$

When F is linear, for instance $F\left(E\right)=F_{\infty }\;E$, we obtain

(50) $$\tau_{T}\overset{□}{\;T}=\tau_{T}F_{0}D-\left(T-F_{\infty }E\right),\;\tau_{q}\overset{□}{\;q}=-\tau_{q}\kappa \nabla \theta -q,$$

where $F_{0}=F_{\infty }+A^{-1}$, $\kappa =A^{-1}/\theta$.

### 7.2. Upper Convected Maxwell Model

When $F_{\infty }=0$ (F≡0) from (50)_1_, we obtain

$$T+\tau_{T}\overset{□}{\;T}=\tau_{T}A^{-1}D,$$

and assuming $\tau_{T}A^{-1}=\lambda 1⊗1+2\mu I$ (isotropy), we have the following:

$$T+\tau_{T}\overset{□}{\;T}=\lambda \left(\mathrm{tr}\;D\right)1+2\mu D.$$

In the special case ∇·v=0 (incompressibility) we recover the upper-convected Maxwell model:

$$T+\tau_{T}\overset{▽}{\;T}=2\mu D.$$

The corresponding free energy density is given by $\rho \psi =\rho \psi_{0}\left(\theta \right)+\frac{\tau_{T}}{4\mu }{\left|T\right|}^{2}$.

### 7.3. Kelvin–Voigt Model in the Spatial Description

When $\partial_{T}\psi ,\partial_{q}\psi =0$, relations (46) reduce to

$$\left(T-\rho \partial_{E}\psi \right)\cdot D=\theta \sigma^{ET},\;q\cdot \nabla \theta =-\theta^{2}\sigma^{Q}.$$

The latter equation can be handled as in Section 5.1. Applying the procedure exhibited in Section 6.3, the former relation yields

(51) $$T=\rho \partial_{E}\psi +\frac{\theta \sigma^{ET}}{{\left|AD\right|}^{2}}A^{2}D,$$

where A is a symmetric, non-singular fourth-order tensor, possibly parameterized by θ. Let $\rho \partial_{E}\psi =\rho \psi_{0}\left(\theta \right)+\frac{1}{2}F_{0}E\cdot E$ and $\sigma^{ET}=\theta^{-1}{\left|AD\right|}^{2}$. Then, (51) becomes

$$T=F_{0}E+A^{2}D.$$

In the isotropic case, $F_{0}=\lambda_{0}1⊗1+2\mu_{0}I$, $A^{2}=\lambda 1⊗1+2\mu I$, we obtain

$$\mathrm{tr}\;T=\left(3\lambda_{0}+2\mu_{0}\right)\mathrm{tr}\;E+\left(3\lambda +2\mu \right)\mathrm{tr}\;D,\;\mathrm{dev}\;T=2\mu_{0}\;\mathrm{dev}\;E+2\mu \;\mathrm{dev}\;D,$$

### 7.4. Bingham–Norton Visco-Plastic Fluid

Assuming $\partial_{E}\psi =\partial_{q}\psi =0$ and X=0 from (47), we have the following:

$$D=H\;\overset{□}{\;T}+\sigma^{ET}\theta \frac{T}{{\left|T\right|}^{2}},\;H\left(\theta ,T\right)=\frac{\rho }{{\left|T\right|}^{2}}T⊗\partial_{T}\psi .$$

Choosing $\lambda ,\tau_{y}>0$, n∈N∪{0} and

$$\sigma^{ET}=\left\{\begin{array}{cc} 0 & \mathrm{if}\;|T|\leq \tau_{y},\\ \frac{1}{\theta \lambda^{n}}{\left|T\right|}^{n}\left(\left|T\right|-\tau_{y}\right) & \mathrm{otherwise}, \end{array}\right.$$

we recover the Bingham–Norton model in the spatial description. The free energy is given by a generic function ψ=ψ(θ,T). In particular, when

$$\rho \psi =\rho \psi_{0}\left(\theta \right)+\frac{1}{2}\alpha \left(\theta \right){\left|T\right|}^{2},$$

we have the following:

$$H\left(\theta ,T\right)=\frac{\alpha (\theta )}{{\left|T\right|}^{2}}T⊗T,$$

so that H enjoys both the minor and major symmetries. The special case α≡0 leads to the well-known model of a Bingham plastic fluid:

$$D=\left\{\begin{array}{cc} 0 & \mathrm{if}\;|T|\leq \tau_{y},\\ \frac{1}{\mu }\left(1-\frac{\tau_{y}}{\left|T\right|}\right)T & \mathrm{otherwise}, \end{array}\right.$$

where

$$\mu =\frac{\lambda^{n}}{{\left|T\right|}^{n-1}}$$

is referred to as Norton–Hoff nonlinear viscosity; it reduces to a constant value, μ=λ, when n=1.

## 8. Higher-Order Rate Models

Non-Newtonian phenomena exhibited, e.g., by asphalt and some biomaterials show properties that can be associated to different relaxation times or rather to higher-order differential equations. This view is often described by having recourse to the Burgers model [32] or the best known Oldroyd-B fluid model [33].

### 8.1. Burgers Material

While sometimes the model is motivated by rheological analogs [34] and framed within a scheme with intermediate reference configurations [35], here, the model is investigated within the thermodynamic approach developed so far.

Let

T=−p1+S.

The stress S in the Burgers model describes an incompressible fluid and is given by the second-order rate equation

(52) $$S+\lambda \overset{▽}{\;S}+\beta \overset{▽▽}{\;S}=2\mu D+\;\nu \overset{▽}{\;D};$$

We expect that, for a compressible fluid, the same equation should hold with the Oldroyd derivative $\overset{▽}{\;S}$ replaced with the Truesdell derivative $\overset{□}{\;S}$. The thermodynamic consistency of (52) can be proven by having recourse to the counterpart of (52) in the reference configuration.

Let $S_{RR}=JF^{-1}SF^{-T}$ be the corresponding stress in the reference configuration. We let

(53) $$S_{RR}+\lambda {\dot{S}}_{RR}+\beta {\ddot{S}}_{RR}=2\mu \dot{E}+\nu \ddot{E}$$

be the analog of the Burgers model in the referential version. To investigate the thermodynamic consistency of (53), we assume the pressure *p*, the entropy η, and the free energy ψ as functions of the set of variables

$$\Xi_{R}=\left(\theta ,E,S_{RR},\dot{\theta },\dot{E},{\dot{S}}_{RR},\ddot{\theta },\ddot{E},{\ddot{S}}_{RR}\right).$$

The Clausius–Duhem inequality becomes

$$\begin{array}{c} -\rho_{R}\left(\partial_{\theta }\psi +\eta \right)\dot{\theta }+\left(S_{RR}-JpC^{-1}-\rho_{R}\partial_{E}\psi \right)\cdot \dot{E}-\rho_{R}\partial_{S_{RR}}\psi \cdot {\dot{S}}_{RR}-\rho_{R}\partial_{\dot{\theta }}\psi \cdot \ddot{\theta }\;\\ -\rho_{R}\partial_{\dot{E}}\psi \cdot \ddot{E}-\rho_{R}\partial_{{\dot{S}}_{RR}}\psi \cdot {\ddot{S}}_{RR}-\rho_{R}\partial_{\ddot{\theta }}\psi \cdot \dddot{\theta }-\rho_{R}\partial_{\ddot{E}}\psi \cdot \dddot{E}-\rho_{R}\partial_{{\ddot{S}}_{RR}}\psi \cdot {\dddot{S}}_{RR}=J\theta \sigma \geq 0. \end{array}$$

The linearity and arbitrariness of $\dddot{\theta },\dddot{E},{\dddot{S}}_{RR}$ imply that

$$\partial_{\ddot{\theta }}\psi =0,\;\partial_{\ddot{E}}\psi =0,\;\partial_{{\ddot{S}}_{RR}}\psi =0.$$

Assume that *p* and η are independent of $\ddot{\theta }$ and $\dot{\theta }$. Hence, the linearity and arbitrariness of $\ddot{\theta }$, and next of $\dot{\theta }$, imply

$$\partial_{\dot{\theta }}\psi =0,\;\eta =-\partial_{\theta }\psi .$$

Consequently, the inequality simplifies to

(54) $$\left(S_{RR}-JpC^{-1}-\rho_{R}\partial_{E}\psi \right)\cdot \dot{E}-\rho_{R}\partial_{S_{RR}}\psi \cdot {\dot{S}}_{RR}-\rho_{R}\partial_{\dot{E}}\psi \cdot \ddot{E}-\rho_{R}\partial_{{\dot{S}}_{RR}}\psi \cdot {\ddot{S}}_{RR}=J\theta \sigma \geq 0.$$

Further restrictions follow depending on the degrees of arbitrariness. The compressibility of the fluid is described by letting ψ depend on E through $J={\left[\det\left(1+2E\right)\right]}^{1/2}$. Since $J=\rho_{R}/\rho$, the classical relation $p=\rho^{2}\partial_{\rho }\psi$ becomes

$$p=-\rho_{R}\partial_{J}\psi .$$

Now, if ψ depends on E through *J* then

$$\partial_{E}\psi =\partial_{J}\psi \partial_{E}J=2\partial_{J}\psi \partial_{C}J=J\partial_{J}\psi C^{-1},$$

whence

$$JpC^{-1}=-\rho_{R}\partial_{E}\psi ,\;p=-\frac{1}{3J}\;\rho_{R}\mathrm{tr}\;\left(\partial_{E}\psi C\right).$$

Consequently, (54) becomes

(55) $$S_{RR}\cdot \dot{E}-\rho_{R}\partial_{S_{RR}}\psi \cdot {\dot{S}}_{RR}-\rho_{R}\partial_{\dot{E}}\psi \cdot \ddot{E}-\rho_{R}\partial_{{\dot{S}}_{RR}}\psi \cdot {\ddot{S}}_{RR}=J\theta \sigma \geq 0.$$

We now investigate the consistency of the Burgers-like evolution Equation (53) with the reduced inequality (55), where the free energy ψ has the form

$$\psi =\psi (\theta ,J,S_{RR},\dot{E},{\dot{S}}_{RR}).$$

For definiteness, we let β≠0 and consider ${\ddot{S}}_{RR}$ as a function of $S_{RR},{\dot{S}}_{RR},\dot{E},\ddot{E}$. Upon substitution of ${\ddot{S}}_{RR}$ from (53) into (55), we have

$$\begin{array}{cc} \left(S_{RR}-\frac{2\mu \rho_{R}}{\beta }\partial_{{\dot{S}}_{RR}}\psi \right)\cdot \dot{E}+\rho_{R}\left(\frac{\lambda }{\beta }\partial_{{\dot{S}}_{RR}}\psi -\partial_{S_{RR}}\psi \right)\cdot {\dot{S}}_{RR} & \\ -\rho_{R}\left(\partial_{\dot{E}}\psi +\frac{\nu }{\beta }\partial_{{\dot{S}}_{RR}}\psi \right)\cdot \ddot{E}+\frac{\rho_{R}}{\beta }\partial_{{\dot{S}}_{RR}}\psi \cdot S_{RR} & =J\theta \sigma \geq 0. \end{array}$$

The linearity and arbitrariness of $\ddot{E}$ imply

(56) $$\partial_{\dot{E}}\psi =-\frac{\nu }{\beta }\partial_{{\dot{S}}_{RR}}\psi ,$$

and then

(57) $$\left(S_{RR}-\frac{2\mu \rho_{R}}{\beta }\partial_{{\dot{S}}_{RR}}\psi \right)\cdot \dot{E}+\rho_{R}\left(\frac{\lambda }{\beta }\partial_{{\dot{S}}_{RR}}\psi -\partial_{S_{RR}}\psi \right)\cdot {\dot{S}}_{RR}+\frac{\rho_{R}}{\beta }\partial_{{\dot{S}}_{RR}}\psi \cdot S_{RR}=J\theta \sigma \geq 0.$$

Now we select the free energy ψ in a quadratic form,

$$\rho_{R}\psi =\rho_{R}\psi_{0}\left(\theta ,J\right)+\frac{\alpha_{1}}{2}|S_{RR}{|}^{2}+\frac{\alpha_{2}}{2}|{\dot{S}}_{RR}{|}^{2}+\frac{\alpha_{3}}{2}{\left|\dot{E}\right|}^{2}+\gamma_{1}{\dot{S}}_{RR}\cdot S_{RR}+\gamma_{2}S_{RR}\cdot \dot{E}+\gamma_{3}{\dot{S}}_{RR}\cdot \dot{E},$$

whence

$$\begin{array}{cc} \partial_{S_{RR}}\psi & =\alpha_{1}S_{RR}+\gamma_{1}{\dot{S}}_{RR}+\gamma_{2}\dot{E},\;\partial_{{\dot{S}}_{RR}}\psi =\gamma_{1}S_{RR}+\alpha_{2}{\dot{S}}_{RR}+\gamma_{3}\dot{E},\\ \partial_{\dot{E}}\psi & =\gamma_{2}S_{RR}+\gamma_{3}{\dot{S}}_{RR}+\alpha_{3}\dot{E}. \end{array}$$

Upon substitution into (56) and (57) we obtain

(58) $$\gamma_{2}S_{RR}+\gamma_{3}{\dot{S}}_{RR}+\alpha_{3}\dot{E}=-\frac{\nu }{\beta }\left[\gamma_{1}S_{RR}+\alpha_{2}{\dot{S}}_{RR}+\gamma_{3}\dot{E}\right],$$

(59) $$A|S_{RR}{|}^{2}+B|{\dot{S}}_{RR}{|}^{2}+C{\left|\dot{E}\right|}^{2}+D{\dot{S}}_{RR}\cdot S_{RR}+ES_{RR}\cdot \dot{E}+F{\dot{S}}_{RR}\cdot \dot{E}=J\theta \sigma \geq 0,$$

where

$$\begin{array}{c} A=\frac{1}{\beta }\gamma_{1},\;B=\frac{\lambda }{\beta }\alpha_{2}-\gamma_{1},\;C=-\frac{2\mu }{\beta }\gamma_{3},\\ D=\frac{\lambda }{\beta }\gamma_{1}+\frac{1}{\beta }\alpha_{2}-\alpha_{1},\;E=1+\frac{1}{\beta }\gamma_{3}-\frac{2\mu }{\beta }\gamma_{1},\;F=\frac{\lambda }{\beta }\gamma_{3}-\gamma_{2}-\frac{2\mu }{\beta }\alpha_{2}. \end{array}$$

To satisfy inequality (59) for all values of $S_{RR}$, $\dot{E}$ and ${\dot{S}}_{RR}$ we assume A,B,C≥0 and

$$\frac{\lambda }{\beta }\gamma_{1}+\frac{1}{\beta }\alpha_{2}-\alpha_{1}=0,\;\;1+\frac{1}{\beta }\gamma_{3}-\frac{2\mu }{\beta }\gamma_{1}=0,\;\;\frac{\lambda }{\beta }\gamma_{3}-\gamma_{2}-\frac{2\mu }{\beta }\alpha_{2}=0;$$

Moreover, from (58) it follows

$$\gamma_{2}=-\frac{\nu }{\beta }\gamma_{1},\;\gamma_{3}=-\frac{\nu }{\beta }\alpha_{2},\;\alpha_{3}=-\frac{\nu }{\beta }\gamma_{3}.$$

So overall there are 6 equations involving 6 unknowns ($\alpha_{1},\alpha_{2},\alpha_{3},\gamma_{1},\gamma_{2},\gamma_{3}$) and 4 real parameters (β,λ,μ,ν). The unique solution is

$$\begin{array}{c} \gamma_{1}=\frac{\beta (\lambda \nu +2\mu \beta )}{\nu^{2}+2\nu \mu \lambda +4\mu^{2}\beta },\;\gamma_{2}=-\frac{\nu (\lambda \nu +2\mu \beta )}{\nu^{2}+2\nu \mu \lambda +4\mu^{2}\beta },\;\gamma_{3}=-\frac{\beta \nu^{2}}{\nu^{2}+2\nu \mu \lambda +4\mu^{2}\beta },\\ \alpha_{1}=\frac{\nu \lambda^{2}+\nu \beta +2\mu \lambda \beta }{\nu^{2}+2\nu \mu \lambda +4\mu^{2}\beta },\;\alpha_{2}=\frac{\nu \beta^{2}}{\nu^{2}+2\nu \mu \lambda +4\mu^{2}\beta },\;\alpha_{3}=\frac{\nu^{3}}{\nu^{2}+2\nu \mu \lambda +4\mu^{2}\beta }. \end{array}$$

Accordingly

$$A=\frac{1}{\beta }\gamma_{1},\;B=-\frac{2\mu }{\nu }\alpha_{2}=-\frac{2\mu \beta^{2}}{\nu^{2}+2\nu \mu \lambda +4\mu^{2}\beta },\;C=\frac{2\mu \nu^{2}}{\nu^{2}+2\nu \mu \lambda +4\mu^{2}\beta }$$

We stress that B and C cannot be positive at the same time, whatever the sign of μ is. Therefore we are led to considering only two particular cases: ν=0 and μ=0.

If ν=0 then it follows $\gamma_{2}=\gamma_{3}=\alpha_{3}=0$. As a consequence, we have $\alpha_{2}=0$,

$$\gamma_{1}=\frac{\beta }{2\mu },\;\alpha_{1}=\frac{\lambda }{2\mu };$$

since A=1/2μ>0, C=0 and B=−β/2μ, this case is admissible only if μ>0, β<0.

If μ=0 then we obtain C=B=0 and A=λ/ν. This case is admissible if either λ≥0, ν>0 or λ≤0, ν<0.

Since β≠0, then this analysis proves the thermodynamic consistency of Burgers’ model provided that either ν=0, β<0, μ>0 or μ=0 and λ/ν≥0.

It is worth contrasting the restrictions obtained with what arises from an application of the Lemma 1 about a second-order tensor Z,

(60) Z=(Z·N)N+(I−N⊗N)G,

with G being an arbitrary second-order tensor. Here, we observe that, in view of (55), equation

(61) $${\ddot{S}}_{RR}\cdot \rho_{R}\partial_{{\dot{S}}_{RR}}\psi =S_{RR}\cdot \dot{E}-\rho_{R}\partial_{S_{RR}}\psi \cdot {\dot{S}}_{RR}-\rho_{R}\partial_{\dot{E}}\psi \cdot \ddot{E}-J\theta \sigma$$

is consistent with thermodynamics if it holds for any pair of functions ψ,σ of $\theta ,\dot{E},S_{RR},{\dot{S}}_{RR}$ subject to σ≥0. The selection $Z={\ddot{S}}_{RR}$ and $N=\partial_{{\dot{S}}_{RR}}\psi /\left|\partial_{{\dot{S}}_{RR}}\psi \right|$ into (60) together with the exploitation of (61) provide

(62) $${\ddot{S}}_{RR}=\left[S_{RR}\cdot \dot{E}-\rho_{R}\partial_{S_{RR}}\psi \cdot {\dot{S}}_{RR}-\rho_{R}\partial_{\dot{E}}\psi \cdot \ddot{E}-J\theta \sigma \right]\frac{\partial_{{\dot{S}}_{RR}}\psi }{\rho_{R}{\left|\partial_{{\dot{S}}_{RR}}\psi \right|}^{2}}+\left(I-N⊗N\right)G.$$

For definiteness we consider the case ν=0, and then we let

$$\rho_{R}\psi =\rho_{R}\psi_{0}\left(\theta ,J\right)+\frac{\lambda }{4\mu }|S_{RR}{|}^{2}+\frac{\beta }{2\mu }{\dot{S}}_{RR}\cdot S_{RR},\;J\theta \sigma =\frac{1}{2\mu }|S_{RR}{|}^{2}-\frac{\beta }{2\mu }{\left|{\dot{S}}_{RR}\right|}^{2},\;\;\mu >0,\;\beta <0.$$

Accordingly,

$$N:=\frac{\partial_{{\dot{S}}_{RR}}\psi }{|\partial_{{\dot{S}}_{RR}}\psi |}=\frac{S_{RR}}{|S_{RR}|}=:\hat{S},\;\frac{\partial_{{\dot{S}}_{RR}}\psi }{\rho_{R}{\left|\partial_{{\dot{S}}_{RR}}\psi \right|}^{2}}=\frac{2\mu }{\beta }\frac{\hat{S}}{|S_{RR}|},$$

so that (62) becomes

$${\ddot{S}}_{RR}=\left[2\mu \dot{E}\cdot S_{RR}-\lambda {\dot{S}}_{RR}\cdot S_{RR}-\beta |{\dot{S}}_{RR}{|}^{2}-|S_{RR}{|}^{2}+\beta {\left|{\dot{S}}_{RR}\right|}^{2}\right]\frac{1}{\beta }\frac{\hat{S}}{|S_{RR}|}+\left(I-\hat{S}⊗\hat{S}\right)G.$$

and then

$$\beta {\ddot{S}}_{RR}=\left(\hat{S}⊗\hat{S}\right)\left[2\mu \dot{E}-\lambda {\dot{S}}_{RR}\right]-S_{RR}+\beta \left(I-\hat{S}⊗\hat{S}\right)G.$$

Letting $\beta G=2\mu \dot{E}-\lambda {\dot{S}}_{RR}$, we obtain exactly Equation (53) with ν=0.

It is of interest to determine the Eulerian version of the rate-type Equation (53). We know that

$${\dot{S}}_{RR}=JF^{-1}\overset{□}{\;S}F^{-T}.$$

To evaluate ${\ddot{S}}_{RR}$, we observe that

$$\begin{array}{c} {\ddot{S}}_{RR}=(JF^{-1}\overset{□}{\;S}F^{-T}{)}^{˙}=\dot{J}F^{-1}\overset{□}{\;S}F^{-T}+J\dot{\bar{F^{-1}}}\overset{□}{\;S}F^{-T}+JF^{-1}(\overset{□}{\;S}{)}^{˙}F^{-T}+JF^{-1}\overset{□}{\;S}\dot{\bar{F^{-T}}}\\ =JF^{-1}\left[(\overset{□}{\;S}{)}^{˙}-L\overset{□}{\;S}-\overset{□}{\;S}L^{T}+\nabla \cdot v\;\overset{□}{\;S}\right]=JF^{-1}\overset{□□}{\;S}F^{-T}. \end{array}$$

Moreover, since $\dot{E}=F^{T}DF$, then

$$\ddot{E}=(F^{T}DF{)}^{˙}=F^{T}\left(\dot{D}+L^{T}D+DL\right)F=F^{T}\overset{△}{\;D}F,$$

with $\overset{△}{\;D}$ being the Cotter–Rivlin derivative. Substituting ${\dot{S}}_{RR},{\ddot{S}}_{RR},\dot{E},\ddot{E}$ in (53), we obtain

(63) $$S+\lambda \overset{□}{\;S}+\beta \overset{□□}{\;S}=J^{-1}B\left(2\mu D+\nu \overset{△}{\;D}\right)B,$$

where $B=FF^{T}$ is the Finger strain tensor. Alternatively, in light of (13),

$$S+\lambda \overset{□}{\;S}+\beta \overset{□□}{\;S}=J^{-1}B\left(2\mu \overset{△}{\;E}+\nu \overset{△△}{\;E}\right)B.$$

If the fluid is incompressible, then

$$0=\mathrm{tr}\;D=C^{-1}\cdot \dot{E},\;p\mathrm{tr}\;D=pC^{-1}\cdot \dot{E}=0;$$

the power of the pressure vanishes. Consistently, we let $\partial_{E}\psi =0$. The results (56) and (57) hold unchanged while (63) changes to

$$S+\lambda \overset{▽}{\;S}+\beta \overset{▽▽}{\;S}=J^{-1}B\left(2\mu D+\nu \overset{△}{\;D}\right)B,\;\mathrm{tr}\;D=0.$$

Hence, in the linear approximation ($J≃1,B≃1,\overset{▽}{\;D}\;≃\;\overset{△}{\;D}\;≃\;\overset{\circ }{\;D}$), the Burgers model (52) is recovered.

### 8.2. Oldroyd-B Model

This model, often used to describe the flow of viscoelastic fluids, can be regarded as an extension of the upper-convected Maxwell model and represents an incompressible fluid filled with elastic bead and spring dumbbells. In this model, named after J. G. Oldroyd [33], the stress is given by the second-order rate equation

(64) $$T+\lambda \overset{▽}{\;T}=2\mu D+\;\nu \overset{▽}{\;D},\;\mathrm{tr}\;D=0.$$

Letting $\lambda =\lambda_{p}$, $\mu =\mu_{s}+\mu_{p}$, $\nu =2\mu_{s}\lambda_{p}$, we can split (64) into a polymeric (viscoelastic) part separately from the solvent (viscous) part,

$$T=2\mu_{s}D+Y,\;Y+\lambda_{p}\overset{▽}{\;Y}=2\mu_{p}D,\;\mathrm{tr}\;D=0,$$

where $\mu_{s}$ and $\mu_{p}$ denote the viscosity coefficients of the solvent and of the solute, respectively. To prove the thermodynamic consistency of (64), we start from its counterpart in the reference configuration:

(65) $$T_{RR}+\lambda {\dot{T}}_{RR}=2\mu \dot{E}+\nu \ddot{E},$$

where $T_{RR}=JF^{-1}TF^{-T}$, and assume the entropy η and the free energy ψ as functions of the set of variables

$$\Xi_{R}=\left(\theta ,E,T_{RR},\dot{\theta },\dot{E},\ddot{E}\right).$$

The Clausius–Duhem inequality becomes

$$\begin{array}{c} -\rho_{R}\left(\partial_{\theta }\psi +\eta \right)\dot{\theta }+\left(T_{RR}-\rho_{R}\partial_{E}\psi \right)\cdot \dot{E}-\rho_{R}\partial_{T_{RR}}\psi \cdot {\dot{T}}_{RR}\;\\ -\rho_{R}\partial_{\dot{\theta }}\psi \cdot \ddot{\theta }-\rho_{R}\partial_{\dot{E}}\psi \cdot \ddot{E}-\rho_{R}\partial_{\ddot{E}}\psi \cdot \dddot{E}=J\theta \sigma \geq 0. \end{array}$$

Assume that η is independent of $\dot{\theta }$. Hence, the linearity and arbitrariness of $\ddot{E}$, $\ddot{\theta }$, and next of $\dot{\theta }$, imply

$$\partial_{\ddot{E}}\psi =0,\;\partial_{\dot{\theta }}\psi =0,\;\eta =-\partial_{\theta }\psi .$$

For definiteness, we also let the free energy ψ be independent of E. Consequently, the thermodynamic inequality simplifies to

(66) $${\dot{T}}_{RR}\cdot \rho_{R}\partial_{T_{RR}}\psi =T_{RR}\cdot \dot{E}-\rho_{R}\partial_{\dot{E}}\psi \cdot \ddot{E}-J\theta \sigma ,$$

that holds for any pair of functions ψ and σ of the variables $\theta ,\dot{E},T_{RR}$ subject to σ≥0. The selection $Z={\dot{T}}_{RR}$ and $N=\partial_{{\dot{T}}_{RR}}\psi /\left|\partial_{{\dot{T}}_{RR}}\psi \right|$ into the representation formula (60) together with the exploitation of (66) provide

(67) $${\dot{T}}_{RR}=\left[T_{RR}\cdot \dot{E}-\rho_{R}\partial_{\dot{E}}\psi \cdot \ddot{E}-J\theta \sigma \right]\frac{\partial_{T_{RR}}\psi }{\rho_{R}{\left|\partial_{T_{RR}}\psi \right|}^{2}}+\left(I-N⊗N\right)G.$$

Let

$$\rho_{R}\psi =\rho_{R}\psi_{0}\left(\theta \right)+\frac{1}{2(\nu +2\mu \lambda )}|\lambda T_{RR}-\nu \dot{E}{|}^{2},\;J\theta \sigma =\frac{\lambda }{\nu +2\mu \lambda }|T_{RR}{|}^{2}+\frac{2\mu \nu }{\nu +2\mu \lambda }{\left|\dot{E}\right|}^{2},$$

where μ>0 and λν>0, so that we have σ≥0. Since $N=\left(\lambda T_{RR}-\nu \dot{E}\right)/|\lambda T_{RR}-\nu \dot{E}|$, then Equation (67) becomes

$$\lambda {\dot{T}}_{RR}=\left(N⊗N\right)\left[2\mu \dot{E}+\nu \ddot{E}-T_{RR}\right]+\lambda \left(I-N⊗N\right)G,$$

and letting $\lambda G=2\mu \dot{E}+\nu \ddot{E}-T_{RR}$ we obtain exactly the whole Equation (65).

To determine the Eulerian version of the rate-type Equation (65), we follow the same procedure as in the previous subsection, and taking into account the incompressibility condition, we obtain

(68) $$T+\lambda \overset{▽}{\;T}=J^{-1}B\left(2\mu D+\nu \overset{△}{\;D}\right)B,\;\mathrm{tr}\;D=0.$$

In the linear approximation ($J≃1,B≃1,\overset{▽}{\;D}\;≃\;\overset{△}{\;D}\;≃\;\overset{\circ }{\;D}$), Equations (64) and (68) coincide. This proves the thermodynamic consistency of Oldroyd-B model provided only that λ≠0,μ>0.

## 9. Conclusions

Thermodynamically consistent viscoelastic models are established on the basis of the following points. The constitutive equations are essentially represented in the Lagrangian description in terms of the Piola–Kirchhoff stress $T_{RR}$ and the Green–St.Venant strain E. For first-order rate type models, the second-law inequality is then stated in the form

(69) $$\left(\rho_{R}\partial_{E}\psi -T_{RR}\right)\cdot \dot{E}+\rho_{R}\partial_{T_{RR}}\psi \cdot {\dot{T}}_{RR}=-\theta \sigma_{R}\leq 0.$$

The entropy production $\sigma_{R}=J\sigma$ is taken as a constitutive function of the chosen independent variables; a similar treatment is developed for the heat conduction effects. The corresponding second-law inequality in the Eulerian description is found to be

$$\left(\rho \partial_{E}\tilde{\psi }-T\right)\cdot D+\rho \partial_{T}\tilde{\psi }\cdot \overset{□}{\;T}=-\theta \sigma \leq 0,$$

with E being the Eulerian Almansi tensor. This formulation proves profitable in the analysis of the thermodynamic consistency of rate-type equations in the Eulerian description. A more complex second law expression than (69) is involved for higher-order rate type models such as Burgers and Oldroyd-B (see § 8).

We now comment on the main novelty of our approach. If $\dot{E}$ and ${\dot{T}}_{RR}$ are assumed to be independent, then one finds that

$$\partial_{T_{RR}}\psi =0,\;T_{RR}=\rho_{R}\partial_{E}\psi ,\;\sigma_{R}=0.$$

If instead $\dot{E}$ and ${\dot{T}}_{RR}$ are not independent, then we determine the mathematical consequence via Lemma 1. For instance, the selection

$$N=\partial_{T_{RR}}\psi /\left|\partial_{T_{RR}}\psi \right|$$

yields a class of rate-type constitutive equations for the stress in the form

(70) $${\dot{T}}_{RR}=\frac{\left(T_{RR}-\rho_{R}\partial_{E}\psi \right)\cdot \dot{E}}{\rho_{R}\left|\partial_{T_{RR}}\psi \right|}N-\frac{\theta \sigma }{\rho_{R}\left|\partial_{T_{RR}}\psi \right|}N+\left(I-N⊗N\right)G$$

where G is an arbitrary second-order tensor. Equation (70) shows that first-order rate equations, consistent with thermodynamics, are determined by a pair of the free energy ψ and the entropy production σ. As a familiar example, the Maxwell model (2),

$${\dot{T}}_{RR}+\frac{1}{\tau }T_{RR}=G_{0}\dot{E},\;\tau >0,$$

follows by letting

$$\rho_{R}\psi =\rho_{R}\psi_{0}\left(\theta \right)+\frac{1}{2}T_{RR}\cdot G_{0}^{-1}T_{RR},\;\sigma =\frac{1}{\theta \tau }T_{RR}\cdot G_{0}^{-1}T_{RR},$$

where $G_{0}$ is a fully symmetric, positive-definite, fourth-order tensor. Indeed, after replacing σ and $\partial_{T_{RR}}\psi$ in (70), we obtain

$${\dot{T}}_{RR}=\frac{G_{0}^{-1}T_{RR}\cdot \left(G_{0}\dot{E}-\frac{1}{\tau }T_{RR}\right)}{|G_{0}^{-1}T_{RR}{|}^{2}}G_{0}^{-1}T_{RR}+\left(I-\frac{G_{0}^{-1}T_{RR}⊗G_{0}^{-1}T_{RR}}{|G_{0}^{-1}T_{RR}{|}^{2}}\right)G$$

Let $G=G_{0}\dot{E}-\frac{1}{\tau }T_{RR}$. The first and last fractional terms cancel each other out, and we have

$${\dot{T}}_{RR}=G_{0}\dot{E}-\frac{1}{\tau }T_{RR}.$$

Throughout this paper, by a proper selection of the independent variables, we determined constitutive equations in the rate-type form, possibly of higher order than the first. To our mind, this has the technical advantage that the recourse to histories is avoided. Moreover, the role of the entropy production as a constitutive quantity allows for the modelling of further dissipation effects as is the case in hysteretic phenomena [8].

Following the lines developed in this paper, future work will be devoted to phase transitions and hysteresis in shape memory alloys.

## Data Availability

The study did not report any data.
